# A comparison of Project Servator and routine stop and search outcomes in the city of London

**DOI:** 10.1057/s41284-024-00461-1

**Published:** 2025-01-20

**Authors:** Zoe Marchment, Paul Gill

**Affiliations:** Department of Security and Crime Science, UCL, 35 Tavistock Square, London, WC1H 9EZ UK

**Keywords:** Deterrence, Counter terrorism, Stop and search, Suspicious behaviour, Criminal decision making

## Abstract

Project Servator is a strategic method of policing designed to deter, detect and disrupt a wide range of criminal activity including terrorism. This paper is the first empirical study to evaluate the stop and search aspect of Servator deployments. We compare the outcomes of 3488 routine stop and searches and 510 Servator stop and searches to determine whether the stop and search tactics used in this initiative produce more positive outcomes than routine stop and searches. We also compare outcomes of stop and searches that occurred on the basis of suspicious behaviour alone, by ethnicity, and through using a geographically matched sample. Collectively, the results indicate that stop and searches that occur during Servator deployments are more efficient than routine stop and search.

## Introduction

After several years of development by the then Centre for the Protection of National Infrastructure (CPNI, now National Protective Security Authority) and City of London Police, Project Servator deployments began in 2014. Project Servator is a strategic method of policing designed to deter, detect and disrupt a wide range of criminal activity including terrorism (CPNI, 2018). Building upon safety and security plans already in place, Servator deployments increase security presence visibility with the intent of impacting upon criminal decision-making.

Deployments are unpredictable. They can occur at any time of day, any day of the week and last for different durations. They involve both plain-clothed and uniformed officers specially trained to recognise signs of criminal intent. Other resources such as police dogs, Mounted Branch, vehicle checkpoints, Automatic Number Plate Recognition (ANPR) and CCTV support Servator officers. Officers are deployed in populous areas (e.g. shopping centres, tourist attractions and transport hubs) and at busy events. Since 2014, Police Scotland, British Transport Police and the Metropolitan Police service, amongst others, used Servator deployments.

Servator deployments intend to create uncertainty and unpredictability about security arrangements. It has the potential to lead to deter those with criminal intent or amplify their risk-taking behaviours, which in turn may increase the chances of detection. Amplified awareness of the risk of apprehension (through measures such as increased or unpredictable police patrols) may alter an offender’s risk perception. It may increase perceived uncertainty and ambiguity, leading to heightened awareness of detection, and as such induce fear and anxiety in the offender. This leads to subtle non-verbal signs indicative of anxiety and agitation which Servator officers are trained to identify. Servator officers interact with individuals displaying suspicious behaviours to negate or confirm suspicions and conduct a search where grounds appear to be present.

An implementation evaluation of Project Servator, which focused on public and community perspectives, called for more systematic evaluation of Project Servator deployments and communications (Baines 2019). This paper is the first empirical study to evaluate the stop and search aspect of Servator deployments. We compare the outcomes of 3488 routine stop and searches and 510 Servator stop and searches that occurred in the City of London between January, 2015 and October, 2017 to determine whether the stop and search tactics used in this initiative produce more positive outcomes than routine stop and searches. We also compare outcomes of stop and searches that occurred on the basis of suspicious behaviour alone, by ethnicity, and through using a geographically matched sample. Collectively, the results indicate that stop and searches that occur during Servator deployments are more efficient than routine stop and search.

## Theory

This section is divided into two sub-sections. The first section provides a brief overview of the core research areas underpinning Project Servator’s deployment including rational-choice approaches to criminal decision-making and deterrence (and their application to counter-terrorism). The second section provides a quick overview of one of Project Servator’s tactics: Stop and Search.

### Criminal decision-making & deterrence

Research depicts offenders as rational and purposeful decision-makers who are more likely to commit crime if the expected rewards exceed the associated risks and effort. An offender acts in their own self-interest whilst calculating the costs and benefits of each possible alternative, before making a choice that offers the greatest benefit and lowest cost (Cornish and Clarke 2016; Gill et al. 2018). Rationality is subject to limits and is guided by time constraints, uncertainty, emotion, available information and the decision-maker’s abilities (Clarke and Felson 1993; Beauregard et al. 2005). Criminal decision-making therefore exhibits bounded rationality (Simon 1955).

One key strand of criminal decision-making literature focuses upon risk perception. Generally, this line of research analyses how individuals calculate the chances of apprehension, as well as the formal and legal sanctions that may be a consequence of arrest (Apel 2013). It is not necessarily fears of the severity of punishment that impacts deterrence, but rather perceptions of the certainty of detection (Pratt et al 2006). Interview-based research consistently demonstrates that overt thoughts of detection and the possibility of failure are a “serious liability” during both the planning stages and during the criminal act itself (Apel & Nagin 2017:137).

An individual’s perceptions of the likelihood of detection and punishment are not stable. Instead, they are consistently shaped by personal and vicarious experiences and through elements of the crime itself (Matsueda et al. 2006; Lochner 2007). Increased awareness of the risk of apprehension (for example through measures such as increased or unpredictable police patrols) should impact upon the offender’s risk perception. Butler’s (1994) study of shoplifters consistently noted that offenders seek to weigh up how effectively deployed a security measure is rather than whether it is present or not. For example, shoplifters outlined that the way shop guards are deployed, rather than just their simple presence, impacts the ‘mood’ of the offender and heightens the perception of risk. Similarly, Cardone and Hayes (2012) provide evidence of how perceptions of employee attentiveness and indifference impact upon risk perceptions. This variance may increase perceived uncertainty and ambiguity of sanctions and as such induce fear and anxiety in the offender. This in turn may lead to heightened awareness of detection.

Several studies have focused on the way emotion affects subjective interpretations of risk, effort and reward, as well as what this means for the practical task for formulating preventive measures designed to influence the choices of prospective offenders. Studies highlight feelings of fear, nerves, stress, tension, worries, apprehension, anxiety, physical sickness and uncertainty are present for street robbers (Alarid et al. 2009), first time sex offenders (Beauregard and Bouchard 2010), shoplifters (Cardone and Hayes 2012; Carmel-Gilfilen. 2013), thieves (Hochstetler 2002), auto-thieves (Cherbonneau and Copes 2006; Copes and Tewksbury 2011; Jacobs and Cherbonneau 2017; Jacobs & Cherbonneau 2017), burglars (Bennett et al. 1984; Cromwell et al. 1991), card fraudsters (Finch 2011), mugging (Lejeune 1977), armed robbers (Kapardis 1988; Kroese and Staring 1994; Gill 2000) and robbers (Feeney 1986; Hochstetler 2001; Kang and Lee 2013; Walsh 1986; Wiersma 1996; Wright and Decker 1994).

Servator is designed to create an adverse operating environment for those involved in criminality and/or intent on conducting attack planning. A central component of Project Servator is an intense communication strategy designed to magnify the effective nature of the tactic, heightening the offender’s perception that they are likely to be disrupted. Offenders operate with a different mind-set to the normal site user. They have guilty knowledge and are aware that they may be behaving in a way that is out of the norm. These factors make them self-conscious and potentially susceptible to detection. As such, offenders have a natural underlying anxiety or paranoia about being detected which can alter their perception of risk and subsequent behaviour. Cues of surveillance are noticed by individuals (Bateson et al. 2006, 2013; Bourrat et al. 2011; Nettle et al 2013). Adding to this, a longer presence of surveillance cues (such as watchful eyes) and high level of arousal (increased heartbeat) can elevate the intensity of the feeling of being watched (Hesslinger et al. 2017). The resulting anxiety and concern can also produce behaviours that make individuals easier to detect.

A crime is likely to occur when a motivated offender converges with a potentially vulnerable victim at a time and place where guardianship is lacking (Cohen and Felson 1979). The presence of guardians (i.e. police and security, but also ordinary citizens) impacts upon the conscious weighing of risk and benefits. Environments can be manipulated so that the perceived costs of committing crime far outweigh the estimated benefits. One of the central strategies of Servator is to therefore increase the risks for offenders to get caught. Evidence consistently shows that sex offenders, burglars, thieves, robbers and terrorists alike become discouraged from criminal acts because of the presence or intervention of ordinary members of the public or individuals tasked with a security function (Hirschfield 2010; Beauregard & Leclerc 2007). Resultantly Jacobs and Cherbonneau (2016:29) suggest: “Areas rich in natural surveillance produce a substantial risk of observation, and being observed is one step from being noticed.”

One of Project Servator’s aims is to act as a deterrent to terrorists, ensuring that they know that they cannot predict when and where they will find security officials. This creates an adverse operating environment for hostile reconnaissance, which is often a necessity for terrorist attack planning and increases risk to the terrorist in terms of detection. Terrorist decision-making mirrors criminal decision-making (Gill et al. 2018). They seek information on how effectively deployed security is, they keep several targets in mind, they factor in both subjective and objective factors. Finally, fear and nerves negatively impact terrorist decision-making processes in planning and carrying out an attack, mirroring findings related to urban criminal cost–benefit decision-making (Gill et al. 2018).

Although their knowledge of the associated effort, rewards and risks is imperfect, an offender will still maximise utility based on what they do know. An offender’s evaluation of security features at potential targets necessitates hostile reconnaissance. This typically involves active information gathering by the would-be offender on the potential target (i.e. an assessment of the associated procedures and characteristics). Such reconnaissance has been studied across a wide-range of crimes like armed robbery, captive-taking, sexual assault, and car theft (Gill 2000; Daniels et al. 2016; Jacobs and Cherbonneau 2017). Offenders actively seek “windows of vulnerability” in the security system and processes (Walsh 1986). Reconnaissance often involves visiting the target site, maintaining surveillance, considering the possibility of being detected and/or caught and a consideration of how to manage risk (Gill 2000). An awareness of security features of the sites and surrounding areas can often lead to doubts and irregular behaviour. This behaviour increases the risk of the terrorist being detected (Gill et al. 2018).

### Stop and search

One of the main tactics used in Servator is stop and search. Stop and search is one of the most used powers in England and Wales (Bradford 2017), however it remains an understudied area within criminological literature. The most common types of routine searches are those requiring grounds, including *s1 Police and Criminal Evidence Act 1984 (PACE),* used to search for stolen property, items for going equipped to steal, and offensive weapons, the *s23 Misuse of Drugs Act 1971* for controlled substances, and the *s47 Firearms Act 1968* for firearms*.* The primary purpose of these powers is to *‘enable officers to allay or confirm suspicions about individuals without exercising their power of arrest’* (PACE A)*.* Police can search members of the public where there are ‘reasonable grounds for suspicion’ which PACE defines as being ‘*based on facts, information, and/or intelligence’.* Stop and search can also be carried out for both individuals and vehicles where there are no grounds for suspicion.

Stop and search is an investigative tool which also acts as a prevention and deterrence technique. A recently published report by the Youth Endowment Fund (Nair et al 2024) found a reduction in crime where stop and search was implemented or increased, and that this extended to neighbouring areas. This report suggested that intelligence-led stop and search should be prioritised, highlighted the importance of thorough training, and found stop and search to be more effective when it is based on stronger grounds. Stop and search makes offending riskier, but to act as a deterrent the public must be aware of the heightened risk. For Project Servator, the idea is to make the police footprint highly visible and very obvious, but unpredictable in time, location and strategy. Unpredictable timing, type and location of security patrols makes it difficult for the offender to determine a pattern of activity that they can exploit with any confidence. Individuals make decisions and adapt their behaviour based on their environment, adjusting their risk perceptions and consequent actions as a result of knowing about such activity.

Servator officers are trained to identify the subtle, sometimes unconscious, behaviours of the individual that are indicative of stress and anxiety. Individuals displaying these signs are stopped and questioned. If the officers decide that the subject’s subsequent actions or responses warrant a search, they can then use their stop and search powers. It is logical to suggest that this technique leads to a higher level of positive disposals than routine stop and search, where ‘stop and account’ is rarely used. Previous studies on stop and search have often only considered arrest rates, which has the potential to skew results. This study will capture non-arrest disposal outcomes that can also be considered as positive disposals, such as cannabis warnings and penalty notices for disorder (PNDs).

#### H1

Servator deployments will lead to a higher number of positive disposals than routine stop and search.

#### H2

The number of positive disposals for stop and searches based on suspicious/nervous behaviour alone^1^ will be higher for Servator than routine stop and search.

Controversy surrounding the use of stop and search has been widely debated in the UK. Stop and search engagements can have a profound effect on the individuals involved. The manner in which each stop and search encounter is undertaken can have the potential to influence the way in which the public view and support the police and have a detrimental effect of community willingness to engage (Bradford 2015). Routine stop and search has been controversial in relation to race as it has a disproportionate impact on black and ethnic minority communities (Bucke 1997; Clancy et al. 2001; Bowling and Phillips 2007; Quinton 2015; Bradford and Loader 2016). Although there are specific circumstances under which a stop and search can be carried out, there have been notable ethnic disparities. Ethnic minority groups are consistently over-represented (Ariel and Tankebe 2018).

A 2011 Ministry of Justice report revealed that black people were seven times more likely to be searched than white people. After a series of reforms were introduced in 2014 this was reduced to just over four times more likely (Home Office, 2015). Asian people were twice as likely to be searched than white people. Controversy surrounding race and stop and search has been cited as a causal factor of the Brixton riots in 1981 (Scarman 1981), and August 2011 riots across London (Morell et al. 2011). Stereotyping is often subconscious and implicit. Previous studies have also described the disproportionate targeting of British Muslim communities as a consequence of responses to terrorist attacks including successful and unsuccessful plots in London, Glasgow and Manchester. Hargreaves’ (2018) analysis of the UK’s Crime Survey data revealed discrepancies in police searches against Muslim respondents.

Because of the emphasis on specific tactics used by Servator officers, ethnic disparities in stop and search should be less disproportionate. Whilst the area studied in this paper, the City of London, has a low ethnic minority resident population (79% white, compared to 60% white for Greater London), the day-time street population are more ethnically diverse.

#### H3

The proportion of visible ethnic minorities subject to Servator stop and search will be less than that of routine stop and search.

The City of London contains the historic centre and primary central business district of London. It covers just one square mile and has a resident population of around 9000, as well as a transient population of approximately 400,000 daily commuters and thousands of tourists. Differences in the ‘profile’ of who is stopped may be due to differences in the ambient population in the locales where Servator and routine stop and searches typically deploy. Therefore, we will make further comparisons by extracting all routine stop and searches that occurred in the same streets as Servator stop and searches. This will allow us to control for the ambient population.

## Data and analysis

The dataset encompassed a total of 3488 routine stop and searches and 510 Servator stop and searches that occurred in the City of London between January, 2015 and October, 2017. The data used were extracted from officers’ completion of incident reports for both routine and Servator stop and searches, which were compiled and provided by the City of London Police. The City of London police prioritise counter-terrorism and public order in addition to traditional policing due to the high number of events that take place in the area. Stop and search forms are completed every time a stop and search takes place and include the following information: (a) the date and location of the search, (b) the grounds used to justify the search, (c) the power used (d) the search outcome, (e) demographic information about the subject. The possible outcomes of a stop and search included arrest, cannabis warning, other disposal, penalty notice for disorder (PND) and no further action (NFA). Penalty notices for disorder (PNDs) were introduced in The Criminal Justice and Police Act, 2001. A PND is not classified as a criminal conviction but can be issued for recordable offences such as shoplifting, cannabis possession and being drunk and disorderly. Arrests, cannabis warnings, other disposals and PNDs were considered positive disposals. NFAs are cases where there was reasonable suspicion to merit a stop, but no positive detection was found following the search.

Analyses were performed using Pearson’s chi-square (*χ*2) to determine differences/similarities between routine and Servator on categorical variables (positive disposal/NFA).

## Results

### Descriptive statistics

Between January 2015 and October 2017 there were 3488 routine stop and searches and 510 Servator stop and searches in the City of London. 91% of routine stop and search subjects were male, 8% were female, and 1% were classed as ‘unknown’. For Servator, 93.5% of subjects were male, 4.5% female, and 2% unknown.

53.5% of routine stop and searches were made under s1 PACE, 45.5% under s23 Drugs, 1% under s47 Firearms. 1 search was made under s44 Terrorism. For Servator, 29% of stop and searches were made under s1 PACE and 71% under s23 Drugs.

### Stop and search outcomes

First, we compared the outcomes of each case, detailed in Table 1. A higher proportion of Servator stop and searches resulted in positive disposal (63%) than routine stop and search (40.5%), in line with hypothesis 1. More than half of routine stop and searches required no further action. 29.5% resulted in arrest, 5% in a cannabis warning, 0.1% in a PND and 6% in other disposals. For Servator, 37% required no further action. 45% resulted in arrest, 9% in a cannabis warning, 1% in a PND and 8% in other disposals.

**Table 1:** Outcomes of routine and Servator stop and searches

Outcome	Routine (*n* = 3488)	Project servator (*n* = 510)
Arrest	29.5%	45%
Cannabis warning	5%	9%
Other disposal	6%	8%
PND issued	0.1%	1%
*(Total positive disposal)*	*(40.5%)*	*(63%)*
NFA	59.5%	37%

A chi-square test of independence was conducted. A highly significant association was found between type of stop and search and outcome (positive disposal or NFA), χ(1) = 97.076, *p* < 0.001. Based on the odds ratio, a positive disposal was 2.57 times more likely for Servator than routine stop and search, providing further support for hypothesis 1.

### Outcomes of searches based on suspicious behaviour

The next analyses focused on comparing Servator (*n* = 175) and routine (*n *= 1098) stop and searches using a subgroup of the main data containing only the cases that were based on suspicious behaviour alone (i.e. not drug sight/smell, ANPR alerts, witnessed theft etc.).

A chi-square test of independence was conducted on stop and searches that were based on suspicious behaviour alone to see if type of stop and search was associated with outcome type for this subgroup. As with the full dataset, a highly significant association was found between outcome and type of stop and search, χ(1) = 66.976, *p* < 0.001. Based on the odds ratio, a positive outcome was 3.7 times more likely for Servator stop and search than routine stop and search. This is in line with hypothesis 2.

### Ethnicity

Further analyses were run to examine associations between outcome and ethnicity. 19% of individuals subject to routine stop and search were Asian. 21.65% were Black, 0.75% were Chinese, Japanese or South East Asian, 2% were Middle Eastern and 54% were White. 2.5% were unknown or not recorded. For Servator, 15% of subjects were Asian, 15% were Black, 0.5% were Chinese, Japanese or South East Asian, 1.5% were Middle Eastern and 66% were White. 2% were unknown or unrecorded.

There was a significant association between type of stop and search and outcomes (χ(1) = 12.282, *p* < 0.001). For ethnic minority cases, a positive outcome was 1.78 times more likely for Servator than routine stop and search. This is in line with hypothesis 3.

### Matched sample

To control for the ambient population, further analyses were run using searches that occurred on streets where both routine and Servator deployments took place (*n* = 79). This sample included 491 Servator searches and 1858 routine searches. There was a significant association between type of stop and search and outcomes (χ(1) = 69.22, *p* < 0.001). A positive outcome was 2.37 times more likely for Servator than routine stop and search.

Using cases within the matched sample based on suspicious behaviour alone (Servator *n* = 212; routine *n* = 561), there was also a significant association between type of stop and search and outcomes (χ(1) = 86.65, *p* < 0.001). A positive outcome was 4.69 times more likely for Servator than routine stop and search.

## Discussion and conclusion

The aim of this study was to ascertain whether there was a difference in performance between routine stop and search and Servator stop and search. Collectively, the results indicate that Servator deployments could be more efficient than routine stop and search. Generally, a higher proportion of Servator stop and searches resulted in positive disposal than routine stop and search. There was a substantial difference in use of legislation for stop and search. Over 70% of Servator stop and searches were made under Misuse of Drugs legislation. This variance may indicate that those carrying concealed drugs are likely to display traits noticeable to Servator officers trained in behavioural detection, leading to further engagement and an eventual search. This suggests that the inclusion of plain-clothed officers and Servator behavioural detection training may be particularly useful to identify those carrying concealed drugs.

Using the complete dataset, we found that a positive disposal was more than twice as likely for Servator than routine stop and search. The efficacy of Servator was further reflected in the analyses that focused on individuals stopped because of suspicious behaviour only. These analyses found that a positive outcome was almost four times more likely for Servator.

To control for variations in the ambient population, further analyses were run using searches that occurred on streets where both routine and Servator deployments took place. A positive outcome was 2.37 times more likely for Servator than routine stop and search. Using this matched sample for cases based on suspicious behaviour alone, a positive outcome was almost five times more likely for Servator than routine stop and search.

These findings indicate that the behavioural detection training given to each Servator officer may be effective. However, it should be noted that the purpose of the present study is not to assess the validity of whether the right suspicious behaviours are being utilised or whether there is validity in spotting suspicious behaviours. More research related to this is needed.

Public support for stop and search is conditional. There are potential unintended negative consequences of stop and search, which have been widely recognised in the policing literature. Rates of successful stops have been persistently low. It should be well targeted, fair and respectful (Centrex, 2005). Quinton et al (2000) highlight the importance of intelligence on stop and search with the aim of improving targeting and enhancing fairness. Fewer individuals were subject to no further action following a Servator stop and search. This has the potential to increase public confidence and trust in the police. Poor handling of stop and search encounters can lead to negativity towards police (Bradford 2015). It is clear from the reviewed literature that negative responses to stop and search practices resonate more prominently than positive attitudes. The proportion of visible ethnic minorities subject to Servator stop and search was less than that of routine stop and search. For ethnic minority cases, a positive outcome was almost twice as likely for Servator than routine stop and search. The more proportionate use of stop and search, which has always been seen as controversial, can support the way police are perceived by the public and be a step forward in repairing justifiable grievances. Police are much more effective in preventing and detecting crime with the assistance and support of the public.

This study is subject to some limitations. Only data from the City of London were included, which covers a very small geographical area. Further testing should be carried out for other areas with different demographics and different settings such as events, transport hubs, sporting venues, etc*.* The Servator sample used in this study is relatively small, so the inclusion of other areas would also help improve the statistical validity. The data used were based on police reporting. Under-recording of incidents is expected in official police data (Bland et al. 2000; Miller 2010). Further, the details recorded may not be completely accurate. The implications of this for this study are unclear.

It would have been preferable to compare the Servator data with routine ‘stop and accounts’. However, this latter measure is rarely used, and data pertaining to this stopped being collected in 2011. The concept of reasonable suspicion is interpreted widely by police officers in practice, clear differences in interpretation existing within and between police forces (Bowling and Phillips 2007). Grounds for reasonable suspicion are subjective and interpretation is dependent on the officer and context (Waddington 1999). Previous research has shown differing thresholds for officers’ execution of routine stop and search. Some consider grounds for a search to be at a similar level to those for an arrest, whereas others consider the threshold to be much lower (Quinton 2011; Quinton and Packham 2016).

Some cost–benefit analysis should be incorporated in future research. The cost of training the Servator tactic and undertaking Servator deployments must be balanced against the cost of potential harm for each crime that is prevented. However, this assessment is difficult. Research that could further enhance the behavioural detection training may be needed. Studies looking at the behavioural cues in risky situations, especially in a criminal context, are needed to further understand how suspicious behaviour can be detected. In their experimental study of behavioural cues in “suspects” versus “non-suspects”, Jian et al (2006) found that those who had a “suspicious” intent behaved significantly different from those who did not. Suspects engaged in behaviours, such as moving away from a target before returning back, to hide their deceptive intention.

Although not the aim of the current study, future research could assess the impact of Project Servator deployments on criminals or the general public. Surveys could be used to measure how effective the presence of Servator deployments are in affecting the behaviour of both of these groups, i.e. the extent to which a criminal believes they would be detected in the presence of a deployment and how reassuring or disconcerting deployments are to the general public. Servator is designed to be a reassuring presence for the public, however increased police activity may be alarming for some.

In conclusion, the findings from this study suggest that further implementation of Servator deployments should be considered. It is clear that the stop and search component of this initiative is more effective than routine stop and search, with positive outcomes being significantly higher for Servator. Future use of this tactic could play a key role in disrupting individuals with criminal intent.

## Footnotes

1 *(i.e. not drug sight/smell, ANPR alerts, theft *etc.*).*
